# End-users’ perspectives on factors affecting implementation and utilization of the Iranian electronic health record system: a qualitative study in a developing country

**DOI:** 10.1186/s12913-023-10033-5

**Published:** 2023-10-05

**Authors:** Hajar Abbasi, Bahlol Rahimi, Mohamad Jebraeily, Aynaz Nourani

**Affiliations:** 1 Student Research Committee, Urmia University of Medical Sciences, Urmia, Iran; 2 Health and Biomedical Informatics Research Center, Urmia University of Medical Sciences, Urmia, Iran; 3 Department of Health Information Technology, School of Allied Medical Sciences, Urmia University of Medical Sciences, Urmia, Iran

**Keywords:** Health information system, End-user, Qualitative study, Electronic Health Records and systems, Clinical information system

## Abstract

**Background:**

As one of the most important information technologies for storing, managing, and exchanging health information, the electronic health record (EHR) plays a major role in the health system. However, these systems in developing countries have been associated with multidimensional issues. The purpose of the present study was the assessment of nonclinical end-users’ points of view on the implementation and utilization of the Iranian electronic health record system.

**Methods:**

This was a large qualitative study conducted in 2021 for 7 months from February to August. In this study, data were collected through in-depth semi-structured interviews with 70 non-clinical end-users in 22 public and six private hospitals of West Azerbaijan province in Iran. To analyze the data, the thematic analysis method was used.

**Results:**

The study results indicated that technical, human, cultural, managerial, and financial readiness are the most important factors affecting the implementation of EHRs in Iran. Among the mentioned factors, technical and human readiness were emphasized more by the users. Also, technical, organizational, human, and managerial factors were identified as factors influencing EHRs utilization, and technical and organizational factors had a stronger role in the system utilization.

**Conclusions:**

According to the results, several factors influence EHR implementation and adequate utilization in Iran. To achieve the predetermined goals of this system, implementation issues and problems of using the system should be considered and solved.

**Supplementary Information:**

The online version contains supplementary material available at 10.1186/s12913-023-10033-5.

## Background

Information technology has made a great evolution in the healthcare system of different countries [1]. Transitioning from manual and paper-based clinical and non-clinical processes to electronic and computer-based processes has resulted in improving the quality of service delivery, satisfaction of the stakeholders, productivity of the health system, and ultimately improvement the public health [2–4]. The Health Information Technology for Economic and Clinical Health (HITECH) Act has clearly emphasized the role of Health Information Technology (HIT), especially Electronic Health Records (EHRs), in the reform of the health system and achieving its main goal, which is the promotion of public health [5].

The EHR is one of the most important health information technologies, which as a repository of clinical and non-clinical data, stores people’s health data from before birth to after death [6]. These valuable data are used for various clinical, managerial, educational, research, and health policy-making purposes. Without such data, effective and timely decisions cannot be made to solve people’s health problems as well as health management issues at the organizational, regional, and national levels [7, 8].

After proving the benefits of EHRs and the successful experiences of capitalist and leading countries, many countries prioritized the design, implementation, and utilization of this technology in their health transformation plans [9]. However, not all countries (especially developing countries) have had successful experiences in the execution of this technology [10]. Although many countries are looking to others as potential models, the lack of consistent terminology and approach has made cross-national comparisons and learning difficult [11]. Studies show that cultural, managerial, technical, human, financial, and organizational issues can affect the success or failure of implementing EHRs projects [9]. These issues can be different in developed and developing countries and have various degrees of severity in terms of impact on the success or failure of EHRs [12]. For many reasons, developing countries take a longer way to full and successful system implementation. Despite, some progress that has already been made regarding EHR implementation in developing countries, sustainability, and widespread adoption remains elusive [4]. The study conducted by Kumar et al. shows that the implementation and use of electronic health records at the national level in developing and low-income countries such as India, Sierra Leone, and Malawi is influenced by several factors. They considered one of the important factors in this failure to be the problem of electronic file integration with the health care system of these countries. However, Kumar believes that the growth of this technology in the context of the health system of developing countries should be continuous [13]. As a developing country, Iran has well understood the benefits of using information technology, especially EHRs, in its health system and has included the development and expansion of this technology in its national development plan [9].

The Iranian electronic health record system (SEPAS acronym in Iran) is a system that was developed to collect, maintain, and present the health information of Iranian citizens for clinical and non-clinical purposes [14, 15]. The predetermined goals of this system include: first creating the necessary platform for providing advanced electronic health services through EHR, second monitoring the quality of health services, third managing the country’s health system based on accurate and integrated information, fourth increasing the speed and quality of production of medical and biological knowledge, and fifth equitable distribution of health resources [15]. In 2010, this system started working as a pilot project and is currently used in all public and private hospitals in Iran to collect and exchange data with the Ministry of Health [16]. The data gathered in this system is limited to patients’ demographic information, physician information, disease codes, codes for diagnostic and treatment procedures codes, insurance data, and causes of death codes [15]. Contrary to the predetermined goals and the passage of about 12 years, the system utilization is limited to monitoring, management, and policy-making, and it is rarely used in the clinical field for diagnosis and treatment. Also, according to the type of data that is collected in the system, the users of this system are limited to non-clinical staff working in hospitals, such as health information technology staff, discharge department staff, and information technology staff. Currently, physicians, nurses, and other specialists involved in the diagnosis and treatment of diseases are not considered users of this system. In general, there is a big gap between the predetermined goals of the SEPAS and its performance, so this system only meets a limited part of the needs of Iran’s health system [14]. Bashiri et al.‘s study shows that the total mean of SEPAS success was not acceptable. SEPAS has not been much successful in providing net benefits like the provision of electronic services which locate patients in the center and improve the delivery of care to them [17]. Also, the study of SheikhTaheri et al. shows that although SEPAS do not play a prominent role in the clinical field, during Covid-19, it has been able to be effective in presenting statistics, decisions-makings, and macro-policies [18].

Considering the huge costs that have been spent on this system, the future vision of the Ministry of Health is to strengthen and improve this system to cover the needs of the health system and achieve the predetermined goals. Therefore, to achieve this goal and implement and utilize SEPAS in all aspects of the health system, examining the views of current users regarding its implementation and utilization can open the way for managers to reform the system and develop a road map for its improvement. For this reason, this research aimed to investigate non-clinical end users’ views of SEPAS regarding the implementation and utilization of this system.

## Methods

This was a phenomenological study that was undertaken in 2021 and before conducting the study, it was reviewed and approved by the review board and ethics committee of Urmia University of Medical Sciences (IR.UMSU.REC.1400.052).

### Setting

The settings of the study were 22 teaching hospitals affiliated with the Urmia University of Medical Sciences and six private hospitals in 16 cities of West Azerbaijan province in Iran. All hospitals were equipped with SEPAS and exchanged data with the Ministry of Health through it.

### Participants

The participants were selected using the purposive sampling method and included staff from discharge units, Health Information Management (HIM) units, and Information Technology (IT) units. A criterion of having at least two years of work experience with SEPAS was considered for all participants. In general, there are three users in any hospital who work with the SEPAS. Therefore, there are 84 users in the 28 hospitals. However, only 70 of them were eligible to participate in the study. It should be noted that the interviewees had no previous acquaintance or relationship with the interviewer.

### Data collection

Data were gathered through in-depth, semi-structured interviews with 70 SEPAS users from February 2021 through August 2021. Before the interviews, an interview guide was created based on the literature review [9, 19–22]. This guide included 20 open and six closed questions. The face and content validity of the interview guide was judged by four experts in the field of medical informatics and health information management. Also, to ensure the clarity and comprehensibility of the questions, initially, four pilot interviews were conducted, and problems in terms of ambiguity of questions were recognized and fixed for final interviews. It should be noted that these four interviews were not considered in the final analysis.

For data collection, the required coordination was done in advance to interview the SEPAS users. Since the users were located at different geographical distances and hospitals within the province, the researcher determined the date, time, and place of the interview by phone before traveling and interviewing. Many interviews (n = 58) were conducted in the workplace of the interviewees by one of the researchers, but due to the spread of COVID-19, some of the interviews (n = 12) were done via voice call. Before performing the interviews, with adequate information about the study objectives, all of the participants signed an informed consent form. With the participants’ permission, the interviews were recorded by a digital recorder. Handwritten notes were taken during face-to-face interviews to capture information that audio recordings could not capture, such as physical gestures, body language, and facial expressions. It should be noted that in most of the interviews, during the interviews, only the researcher and the interviewee were present in the private room. However, due to the limitations of the physical space of the hospitals, in some of the interviews, some employees heard the interviews, but there was no comment. Also, Each person was interviewed only once.

It is important to note that, although data saturation occurred after interviewing half of the interviewees, however, due to the importance of the frequency of factors mentioned by the interviewees, the interview was conducted with the entire considered sample.

### Data analysis

In this study, the thematic analysis approach was performed based on Braun and Clarke’s six phases of data analysis (familiarization with the data, generating initial codes, searching for themes, reviewing themes, defining and naming themes, and generating the report) [23]. Before data analysis, all interview voice files were transcribed verbatim. Then, transcripts were read by one of the researchers several times. The transcripts were clear and legible so they were not returned to the participants.

After acquiring familiarity with the scope, the researchers (n = 3) determined the main concepts and coded them based on the thematic framework. To code the interviews, initially, the common concepts were specified, and then the main categories and subthemes, and themes were determined. Finally, a summary of the results was mailed to the other researchers to check the validity of the results and all of them approved the content. All data analysis was done in MAXQDA software version 10.0.

It should be noted that, due to the large volume of the studied sample and the coincidence of the study with Covid-19, and the difficulty in re-establishing contact with the participants, the results of the analysis were not returned to them.

## Results

In the present study, 70 out of 84 qualified SEPAS users took part in the interviews. The average time length of interviews was 45 min. Table 1 demonstrated the characteristics of the participants.

As illustrated in Table 1, most of the interviewees (n = 36, 51.42%) were female and the highest frequency (n = 39, 55.71%) was related to the age category of 36–45 years. Regarding the education level, the highest frequency (n = 41, 58.57%) was related to those who had a bachelor’s degree. Also, most of the interviewees (n = 45, 64.29%) had a work experience between 10 and 20 years.

**Table 1:** Characteristics of the participants

Variable		Frequency (%)
Gender	Female	36 (51.42%)
	Male	34 (48.58%)
Age	25–35	21 (30.00%)
	36–45	39 (55.71%)
	46–55	10 (14.29%)
Education level	Diploma	1 (1.43%)
	Associate	1 (1.43%)
	Bachelor	41 (58.57%)
	Master	27 (38.57%)
	Ph.D	0 (0.00%)
Job	HIM* staffs	25 (35.71%)
	IT** - staffs	23 (32.86%)
	Discharge staffs	22 (31.43%)
Work experience (year)	2–9	15 (21.42%)
	10–20	45 (64.29%)
	21–28	10 (14.29%)

* HIM (Health Information Management)

** IT (Information Technology)

**Fig. 1:**
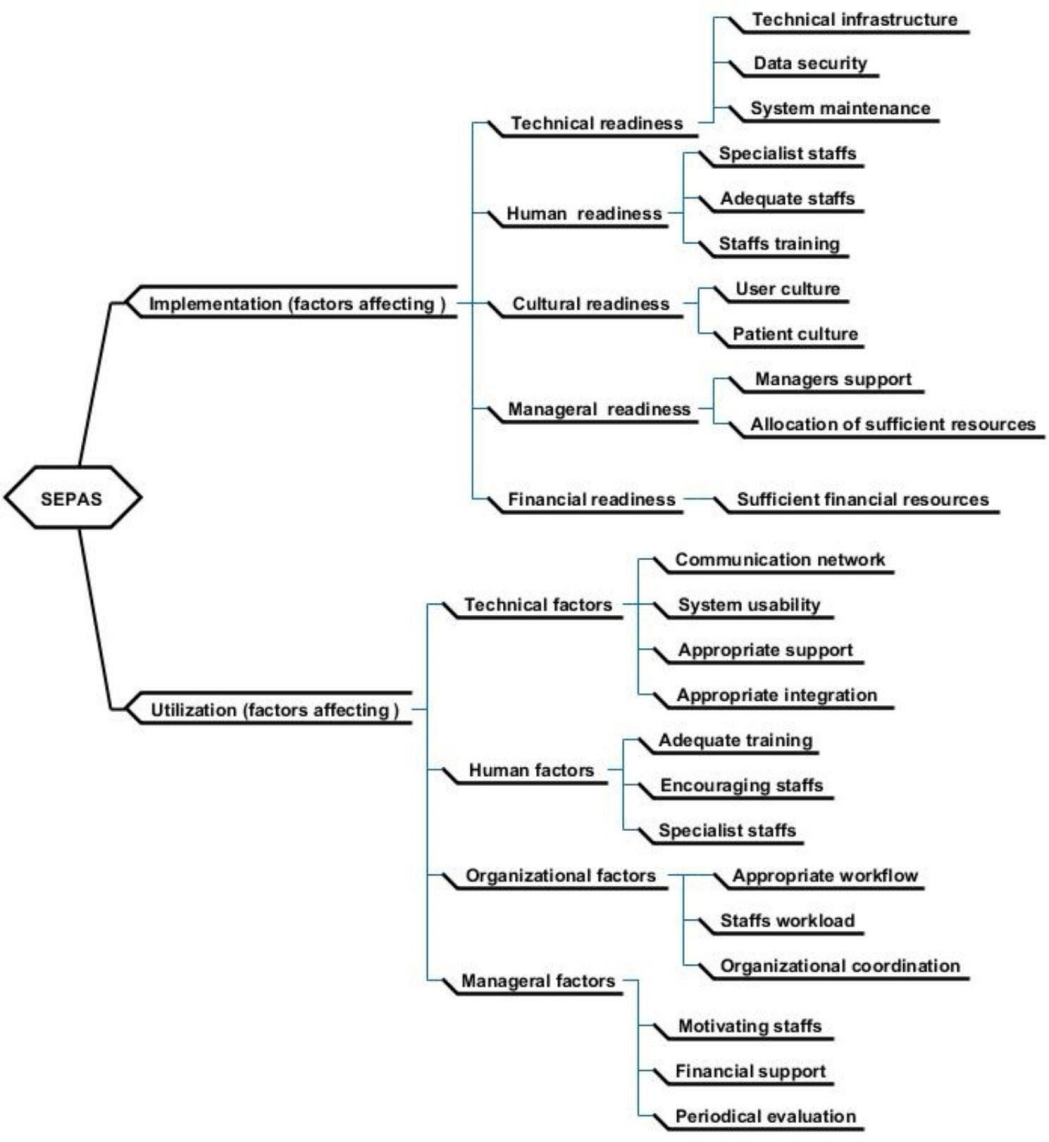
Factors affecting the implementation and utilization of SEPAS

The two main themes extracted from the qualitative study are summarized in Fig. 1.

### Theme 1: Factors affecting the implementation of SEPAS

From the users’ point of view, the main factors affecting the implementation of SEPAS in five groups including technical, human, cultural, managerial, and financial readiness were debatable.

About 49 users mentioned the impact of technical readiness in SEPAS implementation. Most of the users believed that the technical infrastructure, data security, and system support should have been taken into consideration before implementation. For example, one of the interviewees (Participant 29) noted: “The first issue in the implementation of SEPAS is the readiness of technical infrastructure such as the communication network and the possibility of connecting SEPAS with other information systems. Currently, the implemented system has few facilities to communicate with other information systems, which can usually affect its efficiency… On the other hand, no plan has been developed to support the system or maintain data security.”

Human readiness was another sub-theme raised in this theme. About 37 of the interviewees mentioned the importance of human readiness in the implementation of the SEPAS. The interviewees discussed the three areas of specialist staff, adequate staff, and staff training. In this regard, one of the IT staff (Participant 58) mentioned: “In my opinion, before implementing the system, the users of the system should have been evaluated in terms of expertise and the level of training required to work with the system. Few users can work with the SEPAS.”

Another sub-themes in this theme was cultural readiness. About 21 interviewees mentioned the necessity of cultural readiness before implementing the system. They considered the cultural readiness of users and patients as one of the pillars of the success of the system. Regarding the patient culture, one of the HIM staff (Participant 14) said: “When a patient goes to the hospital for admission, some patients refuse to provide information such as their marital status, job, previous illnesses, etc., because they consider this information unnecessary. In general, it can be said that some citizens and even users still do not have the necessary cultural readiness to implement such a system.”

Another existing sub-theme was managerial readiness. Users (n = 19) believed that it is necessary to prepare the organization’s management to accept the system and adopt the necessary policies to prepare a suitable platform for system implementation. They stated that managers should be aware of the decisions of the Ministry of Health to implement such a system. In this regard, one of the users (Participant 5) said: ”In my opinion, the readiness of the organization’s management should have been assessed before implementation. Managers should be aware of the major policies of the Ministry of Health, national standards, and the decisions of the relevant committee and give sufficient motivation to the users of the system to achieve the goals.”

According to the users (n = 9), financial readiness was one of the other factors that affected the implementation of SEPAS. Users believed that a comprehensive financial plan was not developed for the implementation of SEPAS. So that the non-allocation of necessary financial resources by the organization and the Ministry of Health (at a higher level) has caused the organization to not achieve the predetermined goals in the implementation of the system. In this regard, one of the users (Participant 41) said: “During the implementation of the system, there were many financial disputes between the developer company and the organization (such as the incapability of the organization to pay the fees), some of which have not been resolved yet. These differences have affected the successful implementation of the system.”

**Fig. 2:**
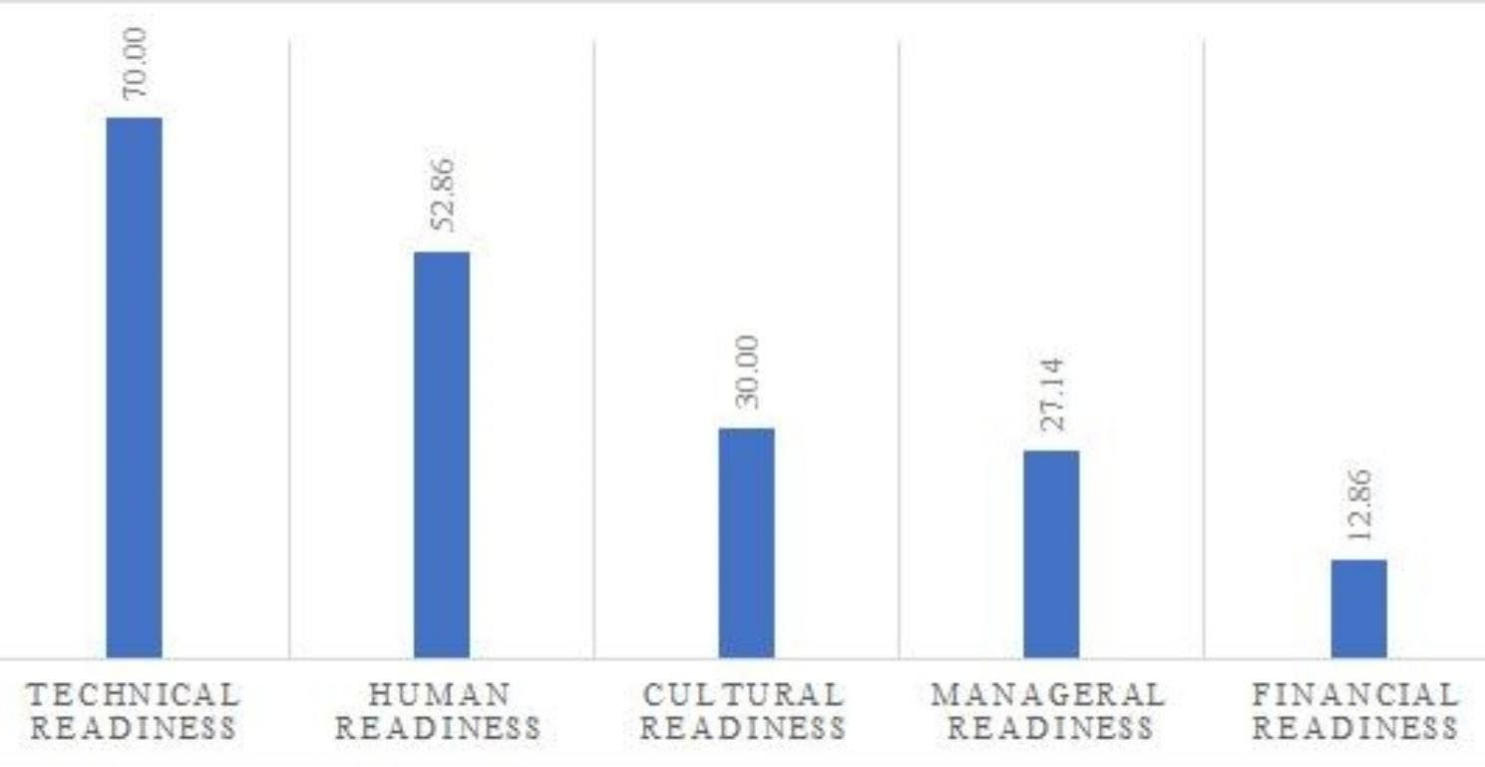
The percentage of emphasis on factors affecting the implementation of SEPAS

Figure 2 illustrates the percentage of emphasis on factors affecting the implementation of SEPAS.

### Theme 2: Factors affecting the utilization of SEPAS

Another main theme was the factors affecting the system utilization. The factors affecting the use of SEPAS were discussed in four groups, including technical, human, organizational, and managerial factors.

About 54 users mentioned the existing technical issues and their impact on the system utilization. The issues raised in four groups, including communication network, system usability, appropriate support, and appropriate integration with other systems, could be discussed. In this regard, the content of the interviewees’ conversations indicated that there is still no communication network with a high-reliability factor, and the occasional system outage causes problems in its utilization. Also, users considered usability as one of the most important reasons for using the system and pointed out the existence of weaknesses in the usability of SEPAS. Lack of sufficient support both in terms of hardware and software was another issue mentioned by users. Finally, some users discussed the importance of integrating the system with other systems, including the hospital information system, and admitted that forcing the use of disintegrated systems can increase the workload, confuse users, and reduce system utilization. For example, one user (Participant 32) said: “A system is used by users when it has the least technical problems. When the usability of the system is low or the communication network is disconnected and connected, it cannot be expected that its use will be maximum. Even if the system is usable and there is no network outage, the technical support team should have enough support for the users… In my opinion, these issues should be resolved so that users can use the system adequately.”

According to users (n = 16), human factors play an important role in using the system. Users’ opinions in this regard were placed in three categories: adequate training, encouraging users, and specialist users. Users believed that appropriate system utilization is achieved when they are given sufficient training before implementing and using the system. Also, users thought that encouraging users can be an important factor in increasing the motivation to use the system. They noted that not receiving rewards and encouragement can discourage users and ignore their efforts. On the other hand, users indicated that system utilization, especially for non-clinical users, should be based on skill and expertise. Some users, such as admission and discharge department users, may have little skill and expertise in working with the SEPAS. Therefore, they may be reluctant to use it. In this regard, one of the HIM users (Participant 62) stated: “Before implementing SEPAS or any other system, you must first evaluate the situation of human resources. Is there an expert to work with the system? Are incentive policies developed for users? Is the system training sufficient? After answering these questions, you can judge the level of system utilization.”

The organizational factor (n = 30) was another sub-theme extracted from the research findings and was divided into the appropriate workflow, staff workload, and organizational coordination. Due to the lack of a suitable and defined work process for using SEPAS, most of the users were reluctant to use this system. Also, some users considered excessive workload as a major reason for not using the system. Some other users considered the lack of proper mechanisms and lack of coordination within the organization as a factor affecting system utilization. In this regard, one of the users (Participant 21) mentioned: “Among the reasons that can discourage me or my colleagues from using the system is the lack of coordination within the organization. When I need to enter the case information into the system, some parts of the case data are not ready due to a lack of coordination among the team members. Better to say, there is no proper workflow in our organizations regarding the use of SEPAS.“

The last sub-theme in this theme was organizational factors. About nine users emphasized the importance of organizational factors such as motivating users, financial support, and periodical evaluations. The users considered the motivational activities of the managers and their financial support of various dimensions as important factors in using the system. Also, to monitor the status of system utilization, users mentioned the impact of periodical evaluations of managers regarding the use of the system. In this regard, one of the users (Participant 53) pointed out: “If a manager monitors my activities in using the system and rewards me for the points I get or reflects my weaknesses, I will work with more effort and motivation. But if there is no evaluation and feedback, there is not much desire to use the system.”

**Fig. 3:**
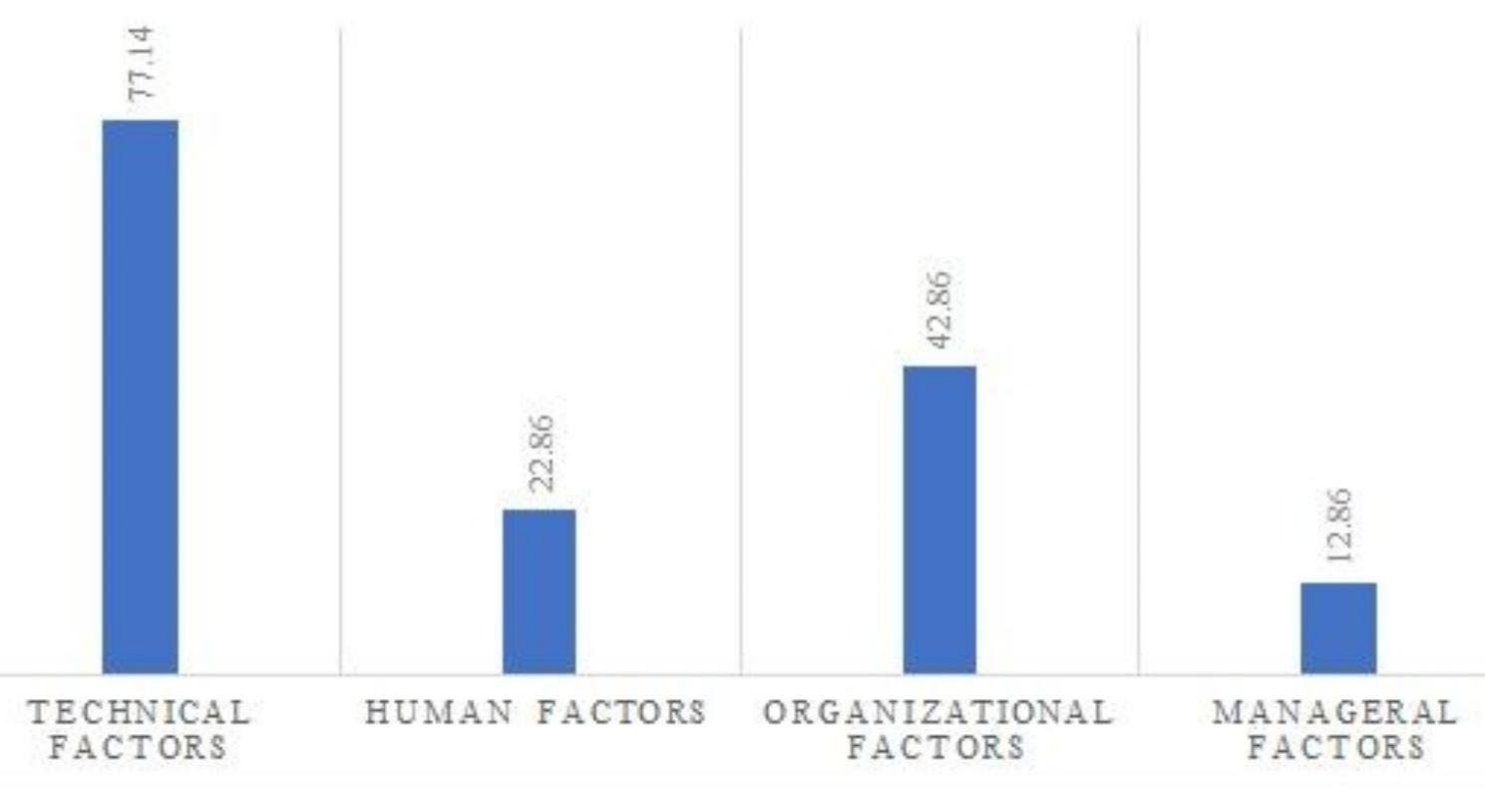
The percentage of emphasis on factors affecting the utilization of SEPAS

Figure 3 showed the percentage of emphasis on factors affecting the utilization of SEPAS.

## Discussion

The EHR is one of the most fundamental information systems, which is considered an information highway for the health system [24]. This system contains all the clinical and non-clinical information of people, which is collected and managed by healthcare organizations at different geographical distances [25]. At the individual level, this information is the basis for medical decisions and financial calculations, and at the organizational, local, and national levels, it is the basis for health management and policymaking [26, 27]. Today, the health system relies heavily on EHRs to manage health data [7]. In different countries, this type of system has reached sufficient maturity after going through its growth path and has been woven into the body of the health system of that country [9, 28]. However, in many other countries, such as Iran, this system is at the beginning or halfway and is in the greatest need of studies that identify the obstacles and problems of the path and regulate the way to reach the goal [9]. Therefore, in this study, the opinion of non-clinical users of Iran’s electronic health record system (SEPAS) was investigated regarding the factors affecting the implementation and use of this system. This system is currently used in all regions of Iran and only by non-clinical users, but in the future, it will be comprehensively used by all clinical and non-clinical users.

The results of the current study indicated that technical, human, cultural, managerial, and financial readiness are among the most important factors affecting the implementation of the SEPAS. Among the mentioned factors, technical and human factors were emphasized more by the users. Lack of attention to information security procedures, weak technical infrastructure, and lack of readiness for system maintenance was among the most important points mentioned in this context. Also, the lack of attention to human resources as system users and their training was considered one of the challenges of system implementation. Similarly, in the study of Abdulai et al. (in Ghana), technical and human readiness were mentioned as the most important factors affecting the implementation of EHRs. In their study, they mentioned the role of planning and financing in providing technical infrastructure and the role of capacity-building workshops for improving the readiness of human resources [29]. Also, Alzghaibi et al. (in Saudi Arabia) pointed out the role of technical and human readiness in the implementation of EHRs on a large scale. They considered insufficient infrastructure, weak connection networks, weak technical support, and insufficient training of end users as important factors in the failure of these systems’ implementation [30]. In Morocco, Parks et al.‘s study showed that human and financial readiness as well as interoperability standards are considered challenges of system implementation [31].

While the studies of developing countries such as the countries of the Middle East have reported technical and human readiness as an important factor in the implementation of EHRs, developed countries have overcome these challenges, and they are looking for solutions to the successful implementation of EHRs integrated with modern technologies such as artificial intelligence [32]. Also, in developed countries with high incomes, comprehensive studies have been conducted regarding the implementation of EHRs with a focus on reducing costs and improving quality [33].

Therefore, it can be acknowledged that to succeed in EHRs implementation, developing countries must pay sufficient attention to the various dimensions of implementation, especially technical and human resources readiness. In these countries, the implementation attitude should be changed from organizational and local to national, and a comprehensive plan should be created to integrate the system with other systems.

Regarding the use of the EHR, the results of the current study indicated that technical, organizational, human, and managerial factors play an important role in the proper utilization of the system. Meanwhile, technical factors (such as communication network, system usability, appropriate support, and integration with other information systems) and organizational factors (such as defined and appropriate workflow, user workload, and organizational coordination) had a stronger role in the use of the system. Similar to the results of the current study, Fennelly et al. emphasized the role of technical, organizational, and human factors in the successful and correct use of EHRs [25]. Afrizal et al. have considered organizational factors such as lack of skilled human resources, insufficient senior management, and lack of interaction between team members as common obstacles to using the system in developing countries; Also, they pointed out that users’ unfamiliarity with the new system and lack of time to use affect the EHRs utilization in developed countries [34]. Like the present study, Srivastava et al. emphasized the relationship between technical problems and system utilization. They reported that technical problems such as outages and weak communication networks can affect system utilization [35]. However, in developed countries, the technical challenges of using the system have rarely been addressed [12]. In general, it can be stated that the technical challenges of using the system, especially the outage of the communication network, often happen in developing countries with low income due to financial and political reasons and can affect the entire system [36].

### Research limitations

Our study has one important limitation. Due to the spread of Coronavirus and the fact that the interviews were conducted at the peak of the epidemic, it was not possible to travel to other provinces. Also, the interview time was shorter than expected. On the other hand, although the experts of the vice-chancellor of the treatment could provide more information about the SEPAS, due to their mission in disease control and management, it was not possible to interview them.

## Conclusion

This qualitative study provided insight into the non-clinical end-users’ perspectives on implementing and utilizing the Iranian Electronic Health Record System and highlighted the issues and challenges. Based on recent results, before implementing health information systems, especially key systems such as EHRs, necessary technical, human, cultural, managerial, and financial readiness should be established in the organization. This readiness will play a significant role in the success of the system after implementation. However, in some developing countries, the time required to reach this readiness can be an obstacle to starting the system. Therefore, some cases are taken into consideration after the initial implementation of the system, and in parallel with the implementation, efforts are made to achieve sufficient preparation. Also, it is very important to consider technical, organizational, human, and managerial factors for the proper use of EHRs by users. These factors can play a decisive role in the success of the system and its achievement of predetermined goals.

The results of the present study, especially the results related to the factors affecting system utilization, can be a guide for SEPAS administrators in order to change the technical, organizational, human, and managerial conditions and re-plan to increase the system utilization. Also, the results related to the factors affecting the implementation of the system can be a guide for other developing countries in order to implement the electronic health record system better.

### Electronic supplementary material

Below is the link to the electronic supplementary material.

Supplementary Material 1

Supplementary Material 2

## Data Availability

All data generated or analyzed during this study are included in this article.

## Abbreviations

EHR: Electronic Health Record
SEPAS: Iranian Electronic Health Record System (acronym in Iran)
HITECH: Health Information Technology for Economic and Clinical Health Act
HIT: Health Information Technology
HIM: Health Information Management
IT: Information Technology
