# Supplementary material for: End-users’ perspectives on factors affecting implementation and utilization of the Iranian electronic health record system: a qualitative study in a developing country
Source: BMC Health Serv Res. 2023 Oct 5;23:1064. doi: 10.1186/s12913-023-10033-5 (PMC10557278; doi:10.1186/s12913-023-10033-5)
Supplement: Supplementary file 1 — Supplementary Material 1 [file 12913_2023_10033_MOESM1_ESM.docx]

**Interview Guide**

| Age: | ……………. | Education level: | ……………. | Work experience: | ……………. |
| --- | --- | --- | --- | --- | --- |
| Gender: | ……………. | Job: | ……………. | Hospital: | ……………. |

1. In your opinion, what are the factors that can affect the implementation of SEPAS?
2. In your opinion, what are the challenges and limitations of the implementation of SEPAS?
3. Do you think technical factors play a role in the implementation of SEPAS?
4. Do you think human factors play a role in the implementation of SEPAS?
5. Do you think cultural factors play a role in the implementation of SEPAS?
6. Do you think managerial factors play a role in the implementation of SEPAS?
7. Do you think financial factors play a role in the implementation of SEPAS?
8. Do you think organizational factors play a role in the implementation of SEPAS?
9. Do you think political factors play a role in the implementation of SEPAS?
10. Do you think social factors play a role in the implementation of SEPAS?
11. In your opinion, what are the challenges and limitations of the utilization of SEPAS?
12. What technical factors can affect your utilization of SEPAS?
13. What human factors can affect your utilization of SEPAS?
14. What cultural factors can affect your utilization of SEPAS?
15. What managerial factors can affect your utilization of SEPAS?
16. What financial factors can affect your utilization of SEPAS?
17. What organizational factors can affect your utilization of SEPAS?
18. What political factors can affect your utilization of SEPAS?
19. What social factors can affect your utilization of SEPAS?
20. Which factors facilitate the utilization of SEPAS?
